# Identification and Characterization of Histone Modification Gene Families and Their Expression Patterns During Pod and Seed Development in Peanut

**DOI:** 10.3390/ijms26062591

**Published:** 2025-03-13

**Authors:** Yingying Chang, Yohannes Gelaye, Ruonan Yao, Ping Yang, Jihua Li, Nian Liu, Li Huang, Xiaojing Zhou, Weigang Chen, Bolun Yu, Huifang Jiang, Boshou Liao, Yong Lei, Huaiyong Luo

**Affiliations:** 1 Key Laboratory of Biology and Genetic Improvement of Oil Crops, Ministry of Agriculture, Oil Crops Research Institute of the Chinese Academy of Agricultural Sciences (CAAS), Wuhan 430062, China; changyingying0908@163.com (Y.C.); yohanes_gelaye@dmu.edu.et (Y.G.); y604987275@163.com (R.Y.); yangping202302@163.com (P.Y.); 19327736306@163.com (J.L.); lnian0531@caas.cn (N.L.); huangli5100@126.com (L.H.); zhouxiaojing@caas.cn (X.Z.); wgchen2015@163.com (W.C.); yubolun@caas.cn (B.Y.); peanutlab@oilcrops.cn (H.J.); lboshou@hotmail.com (B.L.); leiyong@caas.cn (Y.L.); 2 Department of Horticulture, College of Agriculture and Natural Resources, Debre Markos University, Debre Markos P.O. Box 269, Ethiopia

**Keywords:** peanut, histone modification, gene family, pod and seed development, expression profile

## Abstract

Histone methylation and acetylation play potential roles in plant growth and development through various histone modification (HM) genes. However, studies of HM genes are still limited in peanut (*Arachis hypogaea* L.), a globally important oilseed crop. Here, comprehensive identification and investigation of HM genes were performed using the whole genome of peanut, and a total of 207 *AhHMs* encoding 108 histone methyltransferases, 51 histone demethylases, 16 histone acetylases, and 32 histone deacetylases were identified. Detailed analysis of these *AhHMs*, including chromosome locations, gene structures, protein motifs, and protein–protein interactions, was performed. Tandem, segmental, transposed, dispersed, and whole-genome duplications were involved in the evolution and expansion of the HM gene families in peanut. Ka/Ks analysis indicated that the *AhHMs* underwent purifying selection. The expression profiles of the 207 *AhHMs* were investigated during the pod and seed development stages on the basis of the transcriptome sequencing results. Quantitative real-time PCR confirmed that eight *AhHMs* were differentially expressed during pod and seed development. These results provide data support for further studying the epigenetic mechanism of peanut histones, deepen the understanding of seed development, and provide a new direction for the cultivation of more high-yield and high-quality peanut varieties.

## 1. Introduction

Histone modification is an important method of epigenetic regulation in eukaryotes [1]. The dynamically reversible process of histone methylation and demethylation catalyzed by HMTs (histone methyltransferases) and HDMs (histone demethylases), respectively, is the most well-studied type of modification [2]. Acetylation is also dynamically regulated by HATs (histone acetyltransferases) and HDACs (histone deacetylases) [3]. Multiple modifications of histones can form complex regulatory networks that jointly participate in the activation or inhibition of gene expression during various plant developmental processes [4]. Owing to their importance, HM gene families have been investigated in the model plant *Arabidopsis*, model legume *Medicago truncatula* [5], and other important crops, such as sweet orange [6], apple [7], oil palm [8], rubber dandelion [9] and tea [10]. Based on the identified specific domains in these studies, HMTs, HDMs, HATs, and HDACs have been further classified into two, two, four, and three subfamilies, respectively [11]. More specifically, the HMTs were classified into set domain group (SDG) and PRMT (protein arginine methyltransferase) subfamilies; the HDMs were classified into HDMA (SWIRM and C-terminal domain) and JMJ (JmjC domain-containing protein) subfamilies; the HATs were divided into HAC (CBP domain), HAG (GNAT domain), HAF (TAF_II_250 domain), and HAM (MYST domain) subfamilies; and the HDACs were divided into HDA (RPD3/HDA1 superfamily), SRT (silent information regulator 2), and HDT (HD2 families) subfamilies [6,7,8,9,10,11].

HM genes play important roles in plant growth and development, such as seed germination and dormancy, embryonic development, flowering-related processes, fruit development, stress and defense, and hormonal signaling. For example, PRMT5 is a highly conserved arginine methyltransferase protein that regulates plant growth, development, and the environmental stress response [12]. SDG26 and SDG8 are two close HMT homologs that jointly regulate transcription and plant flowering and development [13]. REF6/JMJ12 is a histone H3 lysine 27 (H3K27) demethylase that is required for light initiation of seed germination [14]. LDL1 and LDL2, two lysine-specific demethylases, are involved in histone 3 lysine 4 methylation (H3K4me) of target genes, including flowering loci [15]. Three *Arabidopsis* nucleolar proteins, HDT-I, HDT-II, and HDT-III, play critical roles in the fertility and transcription of rDNAs and rRNA processing-related genes through the regulation of histone acetylation [16]. The HM genes have been shown to be involved in grain development in both rice and wheat [17,18]. Histone H3 lysine 4 trimethylation (H3K4me3) is involved in fatty acid biosynthesis in developing sunflower seeds and affects seed development [19]. The histone modifier gene family plays a role in embryogenesis in oil palm [8], seed development in grapevine [11], and fruit development in sweet orange [6]. Therefore, in peanut, histone modification may also have functions in the regulation of pods and seeds development.

Peanut, also known as groundnut, is an allotetraploid legume originating from the combination of the A and B genomes [20]. It is grown as an important oilseed and cash crop worldwide, playing a critical role in global food and nutrition security. Histone modifications are involved in the regulation of oleosin expression in developing peanut embryos [21]. The overexpression of *AhHDA1* (encoding a histone deacetylase) improved flavonoid, isoflavonoid, and phenylpropanoid metabolism in peanut [22]. Enrichment of H3ac increased the expression of *AhDREB1*, thereby improving plant drought resistance [23]. However, knowledge of HM genes and their functions in peanut is still very limited, and more studies are needed.

Therefore, the present study performed a comprehensive identification and investigation of HM genes in the peanut genome, providing valuable information on their chromosome locations, exon–intron structures, phylogenies, synteny, and interaction relationships. Their expression profiles in different developmental stages of pods and seeds between a pair of near-isogenic lines (NILs) segregating for fruit size were investigated via transcriptome sequencing data, and quantitative real-time PCR (qRT-PCR) was used to validate the identified differentially expressed genes.

## 2. Results and Discussion

### 2.1. Identification and Chromosomes Location of Histone Modification Genes in Peanut

**Figure 1:**
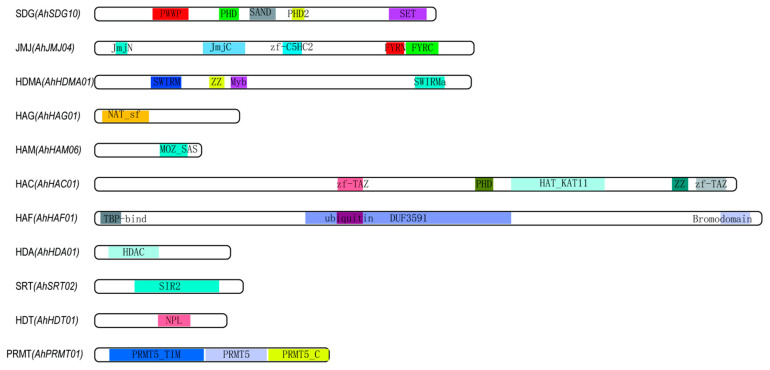
Typical conserved domains for the 11 subfamilies of HM genes in peanut. Distinct domains are indicated by different colored boxes.

In peanut, a total of 207 *AhHMs* were identified, including 108 histone methyltransferases (*AhHMTs*), 51 histone demethylases (*AhHDMs*), 16 histone acetylases (*AhHATs*), and 32 histone deacetylases (*AhHDACs*) (Table S1). Each subfamily (SDGs, PRMTs, HDMAs, JMJs, HAGs, HAMs, HACs, HAFs, HDAs, SRTs, and HDTs) has specific conserved domains to support identification (Figure 1). The 108 *AhHMTs* included 106 SDGs and 2 PRMTs; the 51 *AhHDMs* included 35 JMJs and 16 HDMAs; the 16 *AhHATs* included 4 HAGs, 6 HAMs, 4 HACs, and 2 HAFs; and the 32 *AhHDACs* included 20 HDAs, 4 SRTs, and 8 HDTs. The lengths of the CDSs in the HAC, HAG, HAM, HDA, HDMA, HDT, JMJ, SDG, and SRT subfamilies are 4305–5316, 1442–1773, 447–1338, 273–1542, 1464–5625, 597–1095, 222–5622, 192–7038, and 1233–1716, respectively. In terms of the subfamilies PRMT and HAF, the CDS length of the two *AhPRMTs* is 1941 bp, and that of the two *AhHAFs* is 5529 bp. These *AhHMs* were unevenly distributed among 20 peanut chromosomes, similar to previous reports in other plant genomes [7,24], as summarized in Table 1. Detailed information about these *AhHMs*, including their gene ID, chromosomal position, coding sequence length, protein sequence length, intron number, and exon number, is presented in Table S1.

### 2.2. Gene Structure, Conserved Motif, and Phylogenetic Analysis of Histone Modification Genes

To understand the evolutionary relationships among *AhHM* genes, the exon–intron structure and protein motif of these nine gene families were analyzed. In addition, nine unrooted phylogenetic trees for the 11 subfamilies (PRMT, SDG, HDMA, JMJ, HAC, HAF, HAM, HAG, HDA, HDT, and SRT) were built with *Arabidopsis* and peanut HM proteins. On the basis of these comparisons, each subfamily of the peanut HM proteins was clustered into different groups, with bootstrap support values greater than 50. The members within each group presented similar exon–intron structures and motif combinations.

**Figure 2:**
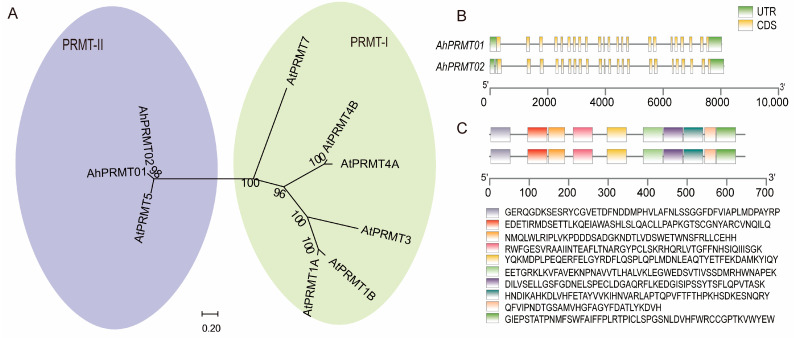
Phylogenetic tree, gene structure, and conserved motif combinations for the PRMT subfamily. (**A**) Phylogenetic tree of PRMT in both peanut and *Arabidopsis*. (**B**) Gene structure of *AhPRMTs*. (**C**) Predicted motifs of proteins encoded by *AhPRMTs*.

The HMTs consisted of the PRMT and SDG subfamilies. The two *AhPRMTs* in the peanut genome (*AhPRMT01* and *AhPRMT02* from the A and B subgenomes, respectively) clustered together with *AtPRMT5* into a group, whereas the other five *AtPRMTs* clustered into another group, indicating that *AhPRMT01* and *AhPRMT02* might be responsible for the function of *AtPRMT5* (Figure 2A). As shown in Figure 2B,C, they had similar gene structure and motif combinations. Among the largest subfamily, SDGs, the 106 and 41 SDGs from peanut and *Arabidopsis*, respectively, were divided into ten groups (SDG-I, SDG-II, SDG-III, SDG-IV, SDG-V, SDG-VI, SDG-VII, SDG-VIII, SDG-IX, and SDG-X) on the basis of their aggregate relationships (Figures S1 and S2). In SDG-III, SDG-VII, SDG-VIII, and SDG-IX, there are twice as many peanut genes as *Arabidopsis* genes because there are two subgenomes in peanut, indicating that genes in these groups are more conserved. However, no peanut gene was classified together with the *Arabidopsis* gene *AtSDG35*, which is also distinct from other *AtSDGs*; thus, SDG-IV consists of only *AtSDG35*.

The HDM family includes two subfamilies, HDMAs and JMJs. Eight of the sixteen *AhHDMAs* were clustered together with four *AtHDMAs* into HDMA-II, whereas the other eight *AhHDMAs* were clustered into HDMA-I without *AtHDMAs* (Figure S3). HDMA-II was more conserved in exon–intron structure and motif combinations than HDMA-I was (Figure S4). The JMJ genes were clustered into four groups, and the groups JMJ-I, JMJ-II, JMJ-III, and JMJ-IV consisted of twenty-one *AhJMJs* and six *AtJMJs*, two *AhJMJs* and five *AtJMJs*, nine *AhJMJs* and six *AtJMJs*, and nine *AhJMJs* and three *AtJMJs*, respectively (Figure S5). However, *AhJMJs* in group JMJ-II had only 2–3 of the 10 identified conserved motifs, indicating that they might have lost their functions during evolution (Figure S6).

Among the four subfamilies for HATs, the HAM subfamily could not be clustered into different groups (Figure S7), and *AhHAM05* and *AhHAM01* shared similar protein motifs (Figure S8). For the HAC subfamily, *AhHAC02* and *AhHAC04* clustered with *AtHAC2* into HAC-II, and *AhHAC01* and *AhHAC03* clustered with four *AtHACs* into HAC-I (Figures S9 and S10). HAG-I had one more motif than HAG-II did (Figure S10). In the HAG subfamily, *AhHAG01* and *AhHAG03* clustered with *AtHAG3* into HAG-I, and *AhHAG02* and *AhHAG04* clustered with *AtHAG2* into group HAG-II (Figures S11 and S12). Therefore, these genes might be orthologs of *AtHAG3* and *AtHAG2* in the two subgenomes of peanut. In terms of the HAF subfamily, *AhHAF01* and *AhHAF02* clustered together into HAF-I, whereas *AtHAF1* and *AtHAF2* clustered together into HAF-II, indicating the divergence of HAF genes during evolution (Figures S13 and S14).

Among the three subfamilies (HDA, HDT, and SRT) for HDACs, HDA was the largest, with twenty *AhHDAs* and twelve *AtHDAs*, which can be divided into three groups, i.e., HDA-I (three *AhHDAs* and *AtHDA2*), HDA-II (eight *AhHDAs* and six *AtHDAs*), and HDA-III (five *AtHDAs* and nine *AhHDAs*) (Figure S15). *AhHDA06* had only three of the ten identified motifs, indicating that its function might have been lost during evolution (Figure S16). The eight *AhHDTs* and four *AtHDTs* were clustered together and could not be divided (Figures S17 and S18). Notably, the four SRT genes from the A (*AhSRT01* and *AhSRT02*) and B (*AhSRT03* and *AhSRT04*) subgenomes were clustered into two groups, and each group had one of the two SRT genes in *Arabidopsis*, suggesting their conservation during evolution (Figure S19). Moreover, *AhSRT01* and *AhSRT03* in SRT-I presented different exon–intron structures and motif combinations than *AhSRT02* and *AhSRT04* did in SRT-II (Figure S20).

For species with limited research foundations, homology analysis is a feasible method to predict the unknown functions of genes, as orthologous genes, which originate from the same common ancestor, often retain the same or similar functions [25,26]. According to the phylogenetic analysis, the peanut and *Arabidopsis* homologs in each subfamily closely clustered together, and *Arabidopsis* genes were homologous to multiple peanut genes.

### 2.3. Synteny and Duplication of Histone Modification Genes

**Figure 3:**
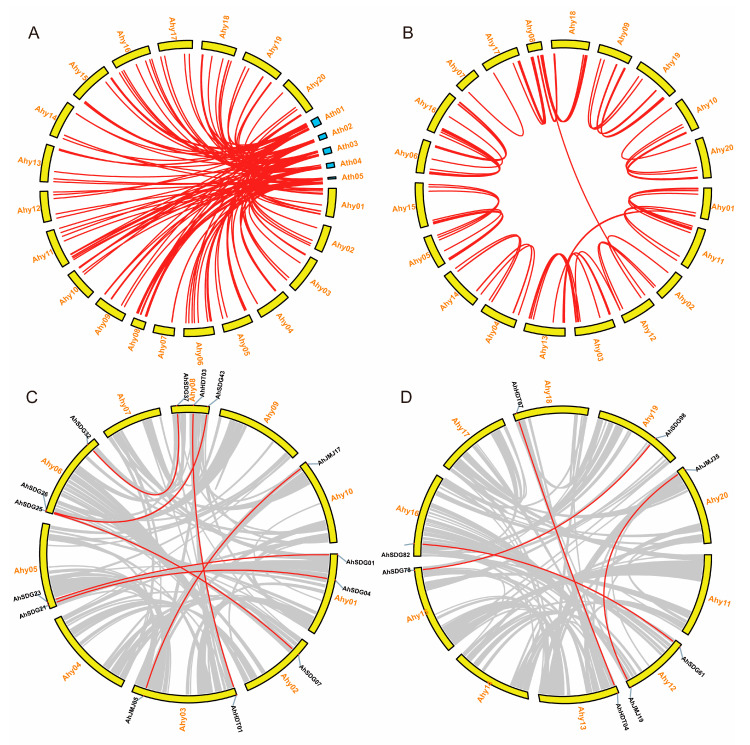
Chromosomal locations and synteny gene pairs of HM genes. (**A**) Chromosomal locations and synteny gene pairs of HM genes between peanut and *Arabidopsis thaliana*; (**B**) chromosomal locations and synteny gene pairs of *AhHMs* between the A and B subgenomes; (**C**) synteny gene pairs of *AhHMs* within the A subgenome; and (**D**) synteny gene pairs of *AhHMs* within the B subgenome. The red lines linked synteny gene pairs of HM genes, and the grey lines linked all synteny gene pairs in peanut. The black fonts indicated the name of HM genes, and the red fonts indicated the name of chromosomal location.

Synteny analysis could identify synteny blocks that would provide more accurate clues about orthologous genes [27]. The roles of HM genes have been well studied in the model plant *Arabidopsis*. Therefore, to gain further insight into the origin and potential functions of HM genes in peanut and to explore their evolutionary history, synteny analysis was performed between peanut and *Arabidopsis*. As shown in Figure 3A, a total of 132 syntenic gene pairs were identified between the HM genes of *Arabidopsis* and peanut (Table S2), which suggests that these genes may have some common origin before speciation, including 87, 8, 7, 2, 2, 14, 8, and 4 Ah-At pairs for SDG, HDMA, JMJ, HAC, HAF, HDA, HDT, and SRT, respectively. However, no syntenic gene pairs were identified for PRMT, HAM, or HAG. The smallest chromosome of peanut (Ahy08) contained the most syntenic gene pairs compared to those of *Arabidopsis*. There were 30 HM genes in *Arabidopsis*, with one syntenic gene pair in each of the two subgenomes of peanut. However, four HM genes (*AtSDG40*, *AtSDG25*, *AtSDG1*, and *AtJMJ11*) in *Arabidopsis* presented syntenic gene pairs only in the A subgenome (Ahy01-Ahy10) or B subgenome (Ahy11-Ahy20), indicating the deletion of these genes in one of the subgenomes during evolution. In addition, 18 HM genes in *Arabidopsis* presented more than two syntenic gene pairs, indicating the occurrence of gene duplication of HM genes after speciation. Take their chromosomal locations into account, the majority of the peanut HM genes presented two copies of the *Arabidopsis* HM genes, which is consistent with the fact that peanut is an allotetraploid legume originating from the combination of the A and B subgenomes [20]. The peanut HM genes with only one copy of the *Arabidopsis* HM genes might have lost their homologs in one of the two subgenomes during evolution.

Synteny analysis of HM genes between subgenomes of peanut was also conducted (Table S3). A total of 79 pairs of syntenic genes were identified between the A and B subgenomes, including 2, 1, 2, 1, 7, 8, 2, 13, 1, 40, and 2 AhA-AhB pairs of *AhHACs*, *AhHAFs*, *AhHAGs*, *AhHAMs*, *AhHDAs*, *AhHDMAs*, *AhHDTs*, *AhJMJs*, *AhPRMTs*, *AhSDGs*, and *AhSRTs*, respectively (Figure 3B). In general, these *AhHMs* were located on corresponding chromosomes in the A and B subgenomes, except for seven pairs, including *AhHDA03* (Ahy01) and *AhHDA15* (Ahy13), *AhJMJ05* (Ahy03) and *AhJMJ19* (Ahy12), *AhSDG43* (Ahy08) and *AhSDG61* (Ahy12), *AhSDG39* (Ahy08) and *AhSDG91* (Ahy17), *AhSDG90* (Ahy17) and *AhSDG37* (Ahy08), *AhHDMA14* (Ahy17) and *AhHDMA06* (Ahy08), and *AhHDMA13* (Ahy17) and *AhHDMA05* (Ahy08), which might be due to segmental exchanges among chromosomes. In addition, the synteny of HM genes within each subgenome of peanut was analyzed. A total of seven (AhA-AhA) and four (AhB-AhB) collinear gene pairs are present within the A (Table S4) and B subgenomes (Table S5), respectively.

**Figure 4:**
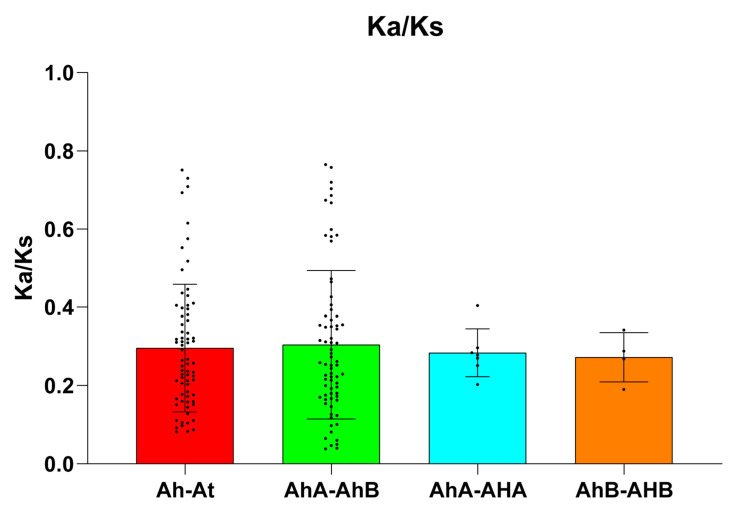
Comparison of Ka/Ks values among different types of syntenic gene pairs. Ah-At represents synteny HM gene pairs between peanut and *Arabidopsis*; AhA-AhB represents synteny HM gene pairs between the A and B subgenomes of peanut; AhA-AhA represents synteny HM gene pairs within the A subgenome of peanut; and AhB-AhB represents synteny HM gene pairs within the B subgenome of peanut.

The average Ka/Ks values of the Ah-At, AhA-AhB, AhA-AhA, and AhB-AhB gene pairs were 0.24, 0.30, 0.28, and 0.27, respectively, indicating that the HM genes underwent purifying selection (Figure 4, Tables S6–S9). On the basis of the Ks value, the Ah-At pairs were estimated to have diverged approximately 161 Mya (Table S6), which is close to the speciation time (~121 Mya). The AhA-AhB pairs were estimated to have diverged approximately 2.61 Mya (Table S7), which is close to the speciation of the A and B genomes in the *Arachis* genus. However, the AhA-AhA and AhB-AhB pairs were estimated to have diverged approximately 50.71 (Table S8) and 53.85 Mya (Table S9), respectively, which is close to the WGD event shared by Fabaceae (~58 MYA). The ratio between Ka and Ks helps to describe the evolutionary process of genes [28]. The results revealed that all the genes underwent purifying selection after duplication and were subjected to selection pressure. Similar results regarding HM genes have been reported in other crop species, such as apple and citrus [7,24]. Therefore, the functions of the 132 peanut HM genes could be dissected on the basis of orthologous genes in *Arabidopsis*.

In order to investigate the origin of nonsynteny homolog gene pairs, the DupGen_finder tool was used to classify the duplication types of homolog pairs. A total of 15 WGD, 65 transposed, 30 dispersed, and 1 proximal duplication events were identified among the 207 HM genes in peanut (Tables S10 and S11), indicating that transposed and dispersed events play dominant roles in gene amplification in HM. Therefore, the nonsynteny homolog gene pairs within the A and B subgenomes might have originated from transposed, dispersed, and proximal replication events that occurred during a long period from 259.87 to 2.63 Mya (Tables S10 and S11). The most duplicated gene pairs were present on chromosome 6 (15), indicating that most duplication events occurred on this chromosome. Notably, some genes were duplicated multiple times, for example, *AhSDG105*, which had six dispersed duplication pairs that occurred from 159.71 to 62.23 Mya (Table S11). In addition, some of the duplicated gene pairs were not clustered tightly together on the evolutionary tree and did not show obvious conservation in terms of gene structure or motif, which might have undergone functional or structural mutations after gene duplication [29].

**Figure 5:**
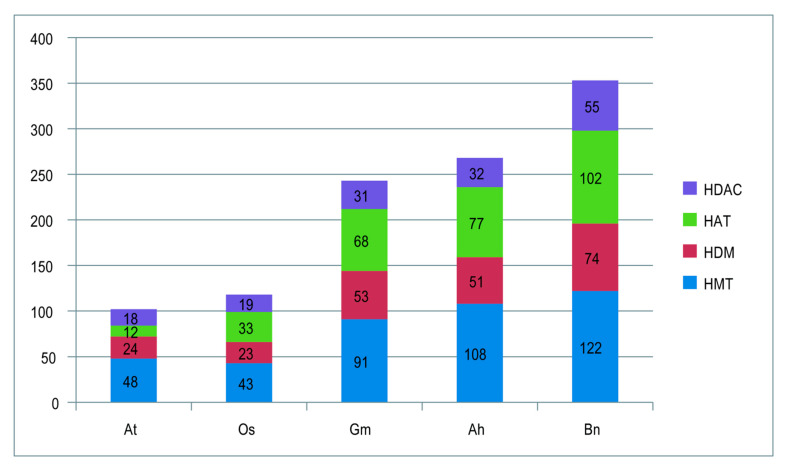
Comparison of the number of histone modification genes in peanut and other plants.

Compared with the 102 and 118 HM genes identified in diploid rice and *Arabidopsis plants*, respectively, 207, 353, and 243 HM genes were identified in peanut (tetraploid), rapeseed (tetraploid), and soybean (diploid) plants (Figure 5). This quantitative differences might be related to the polyploidization events that occurred in peanut and rapeseed [30], as well as the independent WGD event that occurred in soybean ∼10 MYA [31]. Other duplication events such as transposed, dispersed, and proximal events could also contribute the diversification and amplification of genes [32,33,34]. Taken together, the number of histone modification genes could be increased by whole-genome duplication, polyploidization, and other duplication events, and the transposition and dispersion events play a predominant role in the amplification of HM genes in both subgenomes of peanut (Tables S10 and S11).

### 2.4. Potential Interaction of Peanut Histone Modification Genes

**Figure 6:**
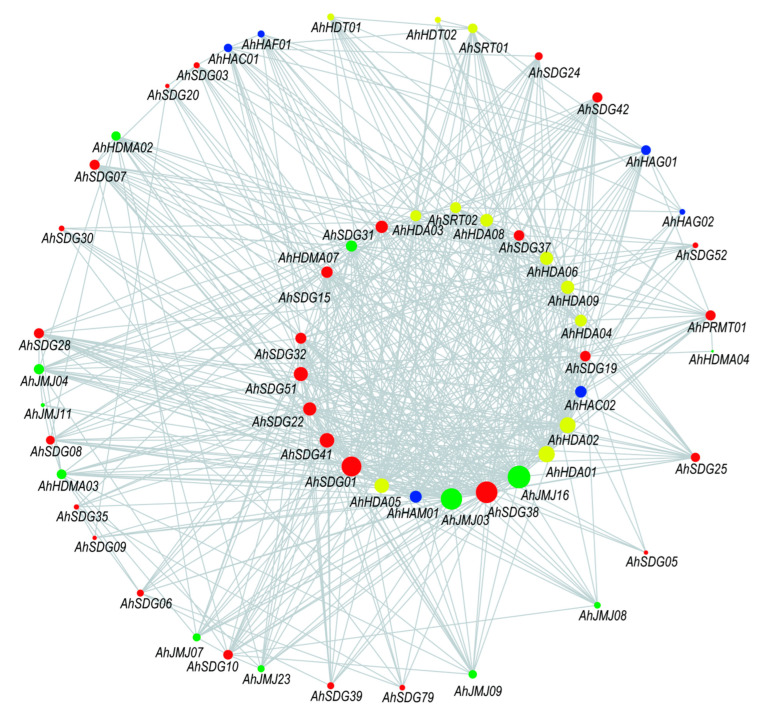
Predicted interactions of histone modification genes in peanut. The circle size indicates the number of interacting genes. The circle colors indicated the groups of genes: red for *AhHMTs*, green for *AhHDMs*, blue for *AhHATs* and yellow for *AhHDACs*.

A total of 538 interactions among 58 *AhHMs* were predicted (Table S12). Among the different subfamilies, SDG has the most interactions (464), whereas HAF has the fewest interactions (12) (Table S13). In HMT, *AhSDG38*, *AhSDG01*, *AhSDG41*, and *AhSDG51* were the top three genes in terms of interactions (Figure 6), and they might interact with 44, 40, 28 and 28 HM genes, respectively. In HDM, the top three genes were *AhJMJ16*, *AhJMJ03*, and *AhHDMA07*, which interact with 46, 43, and 21 genes, respectively. In HAT, *AhHAM01*, *AhHAC02*, and *AhHAG01* might interact with 23, 21, and 18 genes, respectively. In HDAC, *AhHDA01*, *AhHDA02*, and *AhHDA05* were predicted to interact with 33, 32, and 29 genes, respectively. These genes might be candidate hub genes in the interaction network of HM genes in peanut. *AhJMJ16* and *AhJMJ03* are homologs to *AT5G04240* and *AT3G48430*, respectively, which were proved to regulate self-fertility and seed germination in *Arabidopsis* [14,35]. The homolog of *AhJMJ03* is *AT3G4843*, which promotes the activation of genes involved in seed germination in *Arabidopsis* [36]. The corresponding homologs of *AhHDMA07* and *AhHDA01* were *AT1G62830* and *AT4G38130*, respectively, which have a function of regulating seed dormancy in *Arabidopsis* [15,37]. Other gene functions have not been reported in other literature, and further studies are needed.

### 2.5. Identification of Histone Modification Genes Involved in Peanut Pod and Seed Development

**Figure 7:**
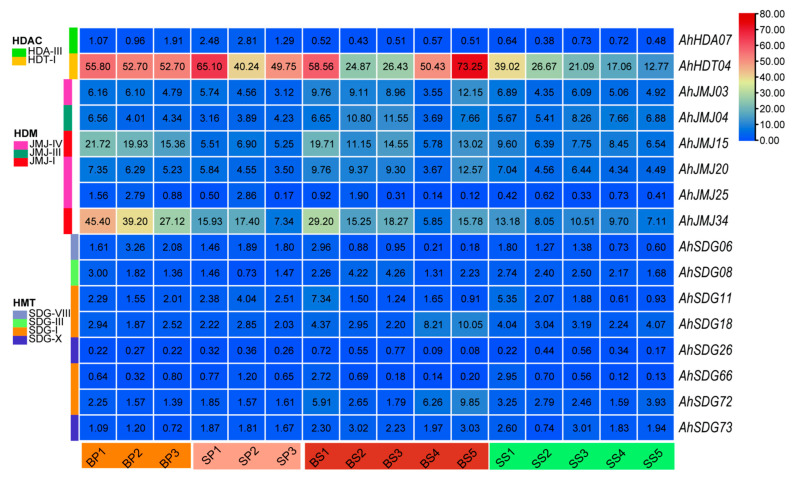
Heatmap of 16 differentially expressed histone modification genes during peanut pod and seed development according to RNA-seq.

**Figure 8:**
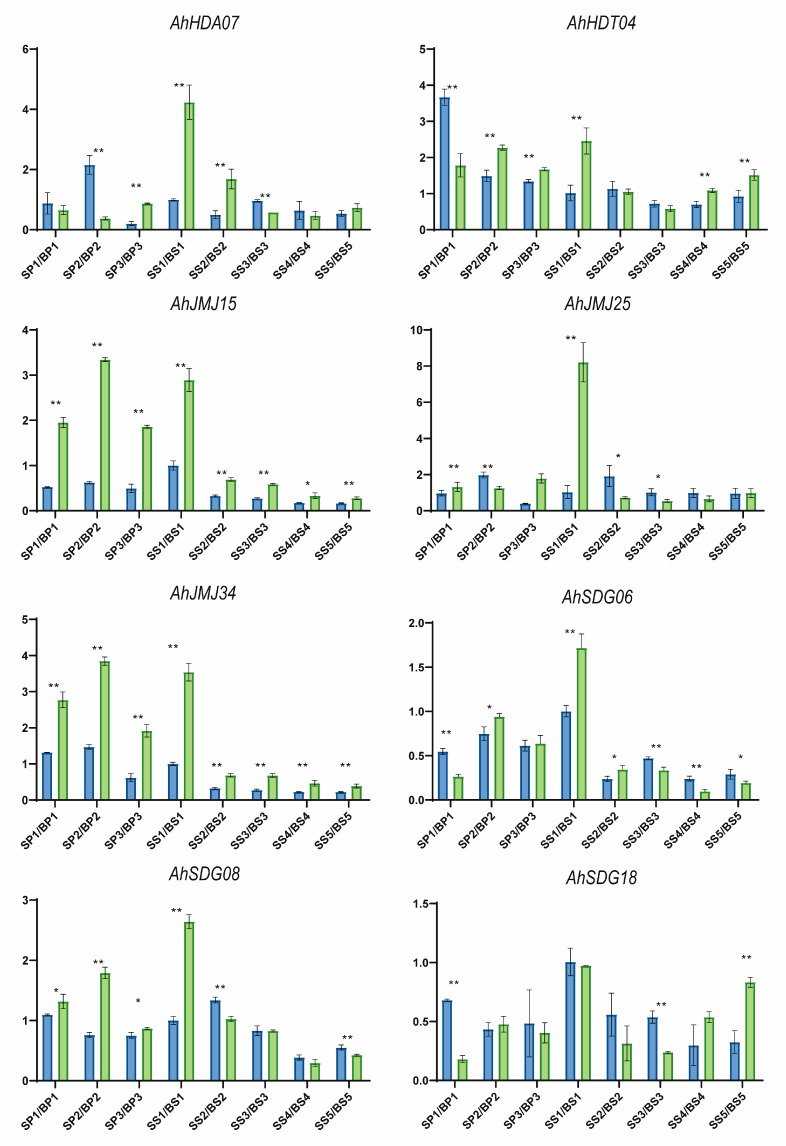
Relative expression levels of eight histone modification genes during peanut pod and seed development validated via qRT-PCR. Each value represents the mean ± standard error of three replicates. Asterisks indicate the corresponding statistical significance (*t* test, * *p* < 0.05, ** *p* < 0.01). The blue bars represent the small-fruit NIL, whereas the green bars represent the large-fruit NIL.

The RNA-seq data were collected from 48 samples collected at three and five different developmental stages from pods and seeds, respectively, from a pair of large (B)- and small (S)-fruit NILs. The average expression of *AhHMs* across all the samples was 5.80, and three genes, *AhHDT03*, *AhHDT04*, and *AhHDT07*, presented RPKM values greater than 40, indicating their importance in the development of pods and seeds in peanut. In contrast, there were 44 HM genes with an RPKM less than one or even equal to zero (Table S14). A total of 7, 9, 6, 6, 3, 3, 18, and 12 significant DEGs (|log_2_(FC)| > 1, *p*-value < 0.01, and FDR < 0.05) were identified for the BP1_vs_SP1, BP2_vs_SP2, BP3_vs_SP3, BS1_vs_SS1, BS2_vs_SS2, BS3_vs_SS3, BS4_vs_SS4, and BS5_vs_SS5 comparisons, respectively. Among them, 16 genes were DEGs at two to five developmental stages (Figure 7, Tables S15 and S16), including the highly expressed gene *AhHDT04*. qRT-PCR primer pairs (Table S17) were successfully designed for 8 of the 16 DEGs. As shown in Figure 8, the expression of the eight genes significantly differed during pod and seed development, indicating that these *AhHMs* are involved in the differential development of pods and seeds between large and small fruit NILs. Notably, the expression of *AhJMJ15* and *AhJMJ34* was greater in the large-fruit NIL than in the small-fruit NIL at all developmental stages (Figure 8).

Histone modifications have been proved to contribute to the regulation of gene expression during fruit developmental processes in crops. For example, the histone modification H3K4me3 enrichment in the *OsMADS1* promoter increased grain width in rice [17]; H3K27me3, H3K4me3, and H3K9ac were involved in the regulation of expression of genes related to starch and storage protein synthesis in wheat endosperm development [18]. Therefore, the 16 differentially expressed *AhHMs*, especially the 8 genes validated by qRT-PCR (*AhHDA07*, *AhHDT04*, *AhJMJ15*, *AhJMJ25*, *AhJMJ34*, *AhSDG06*, *AhSDG08*, and *AhSDG18*), might involve in the regulation of pod and seed size in peanut. Further studies could be conducted to obtain exact information about their gene functions, which would provide insightful information for elucidating the molecular mechanisms of peanut seeds and pods during development.

## 3. Materials and Methods

### 3.1. Identification and Characterization of Histone Modification Genes in Peanut

The HMM profiles of the HM genes (Table 2) were downloaded from the Pfam database [38], and the whole-genome protein sequences of the peanut variety Tifrunner were downloaded from the PeanutBase database (www.peanutbase.org). The HMMER 3.0 tool (HMMER.org) was used to find the candidate HM genes. In addition, the candidate HDT genes were identified via BLASTP (version 2.15.0, https://blast.ncbi.nlm.nih.gov/Blast.cgi) [39] searches with four protein sequences of *Arabidopsis* encoded by *AtHDT1* (*AT3G44750*), *AtHDT2* (*AT5G22650*), *AtHDT3* (*AT5G03740*), and *AtHDT4* (*AT2G27840*), owing to the lack of an HMM profile for HDT in the Pfam database (http://pfam.xfam.org/). The identified peanut HM genes were then named according to their chromosome order. A random gene from each subfamily was selected to illustrate its domain structure and then plotted via EXCEL VBA.

### 3.2. Phylogenetic Analysis of Histone Modification Genes

To illustrate the phylogenetic history and classify the identified HM genes in peanut, genetic trees of HM protein sequences from both *Arabidopsis* (https://www.arabidopsis.org/) and peanut were constructed via the IQ tree method in TBtools software(v2.154, Chengjie Chen, Guangdong, China) [40]. The beautification of the constructed genetic trees was conducted via MEGA 7.0 [41].

### 3.3. Gene Structure and Motif Analysis of Peanut Histone Modification Genes

The gene structure annotation files of the peanut HM genes were also downloaded from the PeanutBase database. The protein sequences of the candidate *AhHMs* were submitted to the MEME website (https://meme-suite.org/meme/tools/meme (accessed on 4 July 2024)) [42] to characterize motif combinations, and at most ten motifs were identified. Schematic diagrams of the gene structure and protein motifs for each subfamily were obtained via TBtools with a genetic tree file, a gene structure annotation file, and a meme file.

### 3.4. Synteny Analysis of Histone Modification Genes

For each subgenome of cultivated peanut, BLASTP (version 2.15.0, https://blast.ncbi.nlm.nih.gov/Blast.cgi) [39] was used for gene similarity analysis against *Arabidopsis*, and MCSCAN [43,44,45] was used to find collinear blocks between the peanut subgenome and the *Arabidopsis* genome. Synteny gene pairs of HM genes were identified from the identified collinearity blocks. TBtools software (v2.154, Chengjie Chen, Guangdong, China) [40] was used to visualize the distribution of *AhHMs* on chromosomes and their collinearity relationships [46]. Synteny analysis of HM genes between the A and B subgenomes of peanut was conducted via the same method. In addition, the DupGen_finder tool was used to classify the duplication types of homolog pairs. Ka/Ks values between gene pairs were calculated in TBtools and illustrated with GraphPad 8.0. The divergence time (T) was estimated as T = Ks/(2 × 8.12 × 10^−9^) million years age (Mya) on the basis of a divergence rate of 8.12 × 10^−9^ synonymous mutations per synonymous locus per year [47].

### 3.5. Interaction Analysis of Peanut Histone Modification Genes

The candidate interactions of HM genes in peanut were predicted according to information in the STRING database for *Arabidopsis* [48]. The overview interaction graph was drawn via Cytoscape 3.7 [49].

### 3.6. Expression Profiling of Histone Modification Genes During Peanut Seed and Pod Development

RNA-seq data, which revealed the gene expression profiles at three and five stages during pod and seed development in a pair of NILs segregating for the identified QTL *qHPWA01.1* [50] controlling pod and seed size, were used to identify *AhHMs* involved in pod and seed development in peanut. The hundred-pod weight for the large-fruit NIL was 206.76 ± 21.69 g, whereas that for the small-fruit NIL was 101.04 ± 16.68 g, indicating a significant difference. Pod samples were collected at the early (P1), middle (P2), and late (P3) developmental stages, and seed samples were collected from the early (S1) to mature (S5) stages. The samples from the large-fruit NIL were denoted BP1, BP2, BP3, BS1, BS2, BS3, BS4, and BS5, respectively, and SP1, SP2, SP3, SS1, SS2, SS3, SS4, and SS5 were used for the small-fruit NIL. Each sample had three replicates. These tissues were used for RNA-seq via the Illumina platform at Igenebook Co. (Wuhan, China). The raw reads were cleaned with cutadapter (version 1.11) and then filtered with the software fastqc (version: 0.11.5) to obtain high-quality reads. The high-quality reads were mapped to the Tifrunner genome with Hisat2 (version: 2.0.1-beta), and duplicated reads were removed. The featureCounts from the Subread package (version 2.0.6, https://subread.sourceforge.net/) was used to quantify the gene expressions with the following parameters: -Q 10–B-C. The mean value of RPKM (Reads Per Kilobase per Million mapped reads) was calculated to represent the expression profiles of *AhHMs*. Differentially expressed *AhHMs* were identified with |log_2_(Fold Change)| > 1, *p*-value < 0.01, and FDR < 0.05 as the criteria [51].

### 3.7. Total RNA Extraction and Quantitative Real-Time PCR Analysis

To validate the expression levels of the identified *AhHMs* that might be involved in pod and seed development in peanuts, RNA was extracted with the FastPure Plant Total RNA Isolation Kit (Polysaccharides & Polyphenolics-rich) (Vazyme, cat: RC 401-01), and the integrity and concentration were detected via a UV spectrophotometer. cDNA was generated with the HiScript^®^ III 1st Strand cDNA Synthesis Kit (+gDNA wiper) (Vazyme, cat: R312). The primers for the target genes were designed via Primer 5.0 software and double-checked via the Oligo 7 and Primer-BLAST methods (https://www.ncbi.nlm.nih.gov/tools/primer-blast/). The specificity of the designed primers was further checked through agarose gel electrophoresis. The quantitative PCR analysis was conducted with the ChamQ Universal SYBR qPCR Master Mix (Vazyme, cat: Q711-02) using the Bio-Rad CFX96 system. The ∆∆Ct method was used to compare the relative expression levels, with β-actin used as the reference gene [52].

## 4. Conclusions

In this study, we identified 207 HM genes belonging to 11 subfamilies in peanut. The chromosome locations, gene structures, motif combinations, phylogenetic and synteny relationships, and protein–protein interactions of these genes were also characterized. Eight candidate HM genes involved in the regulation of pod and seed size in peanut were then identified via RNA-seq and qRT-PCR. Overall, our identification and characterization of HM genes in peanut provide a basis for further characterization of the functions of *AhHMs*, especially in peanut pod and seed development, provide data support for further studying the epigenetic mechanism of peanut histones, deepen the understanding of seed developmental, and provide a new direction for the cultivation of more high-yield and high-quality peanut varieties.

## Figures and Tables

**Table 1:** Distribution of histone modification genes across chromosomes of peanut.

	HMT		HDM		HAT				HDAC			
Chr	SDG	PRMT	JMJ	HDMA	HAG	HAM	HAC	HAF	HDA	HDT	SRT	Total
Chr01	6		1						4		1	12
Chr02	3		1									4
Chr03	3		4	1	2	1			4	1		16
Chr04	7						1					8
Chr05	5		2	2				1				10
Chr06	10		3	1		2			1	1		18
Chr07	2		1									3
Chr08	7	1	1	4					1	1	1	16
Chr09	6		3			1						10
Chr10	5		1				1					7
Chr11	6		1						3		1	11
Chr12	5		1									6
Chr13	3		4	1	2	1			3	2		16
Chr14	5		1				1					7
Chr15	6		3	2				1				12
Chr16	9		3	1					1	1		15
Chr17	3			2					1			6
Chr18	3	1		2					2	2	1	11
Chr19	6		4									10
Chr20	6		1			1	1					9
Total	106	2	35	16	4	6	4	2	20	8	4	207

**Table 2:** HMM accessions used to identify histone modification genes in peanut.

Family Name	Subfamily Name	Accession Number
HMTs	SDGs	PF00856
	PRMTs	PF05185
HDMs	HDMAs	PF04433
	JMJs	PF02373
HATs	HAGs	PF00583
	HAMs	PF01853
	HACs	PF08214
	HAFs	PF09247
HDACs	HDAs	PF00850
	SRTs	PF02146
	HDTs	/

## Data Availability

Data are contained within the article and Supplementary Materials.

## Supplementary Materials

The following supporting information can be downloaded at https://www.mdpi.com/article/10.3390/ijms26062591/s1.
