# Supplementary material for: Identification and Characterization of Histone Modification Gene Families and Their Expression Patterns During Pod and Seed Development in Peanut
Source: Int J Mol Sci. 2025 Mar 13;26(6):2591. doi: 10.3390/ijms26062591 (PMC11942463; doi:10.3390/ijms26062591)
Supplement: Supplementary file 1 [file ijms-26-02591-s001.zip › ijms-3494466-supplementary info(changed).pdf]

Supporting Information

Figure S1 Phylogenetic tree and groups of the SDG subfamily in peanut and *Arabidopsis*

Figure S2 Gene structure and predicted protein motifs of *AhSDGs*

Figure S3 Phylogenetic tree and groups of the HDMA subfamily in peanut and *Arabidopsis*

Figure S4 Gene structure and predicted protein motifs of *AhHDMAs*

Figure S5 Phylogenetic tree and groups of the JMJ subfamily in peanut and *Arabidopsis*

Figure S6 Gene structure and predicted protein motifs of *AhJMJs*

Figure S7 Phylogenetic tree and groups of the HAM subfamily in peanut and *Arabidopsis*

Figure S8 Gene structure and predicted protein motifs of *AhHAMs*

Figure S9 Phylogenetic tree and groups of the HAC subfamily in peanut and *Arabidopsis*

Figure S10 Gene structure and predicted protein motifs of *AhHACs*

Figure S11 Phylogenetic tree and groups of the HAG subfamily in peanut and *Arabidopsis*

Figure S12 Gene structure and predicted protein motifs of *AhHAGs*

Figure S13 Phylogenetic tree and groups of the HAF subfamily in peanut and *Arabidopsis*

Figure S14 Gene structure and predicted protein motifs of *AhHAFs*

Figure S15 Phylogenetic tree and groups of the HDA subfamily in peanut and

Arabidopsis

Figure S16 Gene structure and predicted protein motifs of *AhHDAs*

Figure S17 Phylogenetic tree and groups of the HDT subfamily in peanut and *Arabidopsis*

Figure S18 Gene structure and predicted protein motifs of *AhHDTs*

Figure S19 Phylogenetic tree and groups of the SRT subfamily in peanut and *Arabidopsis*

Figure S20 Gene structure and predicted protein motifs of *AhSRTs*

Supplementary Tables

Table S1 Detailed information for the identified histone modification genes in peanut

Table S2 Histone modification gene pairs within synteny blocks between peanut and *Arabidopsis*

Table S3 Histone modification gene pairs within synteny blocks between the A and B subgenomes of peanut

Table S4 Histone modification gene pairs within synteny blocks within the A subgenome of peanut

Table S5 Histone modification gene pairs within synteny blocks within the B subgenome of peanut

Table S6 Ka/Ks values for syntenic histone modification gene pairs between peanut and *Arabidopsis*

Table S7 Ka/Ks values for syntenic histone modification gene pairs between the A and B subgenomes of peanut

Table S8 Ka/Ks values for syntenic histone modification gene pairs within the A subgenome of peanut

Table S9 Ka/Ks values for syntenic histone modification gene pairs within the B

subgenome of peanut

Table S10 Ka/Ks values for homologous histone modification gene pairs in the A subgenome of peanut

Table S11 Ka/Ks values for homologous histone modification gene pairs in the B subgenome of peanut

Table S12 Identified interaction gene pairs of histone modification genes in peanut

Table S13 Number of interaction nodes for each histone modification gene in peanut

Table S14 Average RPKM values of histone modification genes during peanut pod and seed development

Table S15 The 16 differentially expressed histone modification genes identified via RNA-seq

Table S16 Detailed p-values and FDR values of the 16 differentially expressed histone modification genes identified via RNA-seq

Table S17 qRT–PCR primer information for eight validated histone modification genes
