# Supplementary figures and images for: Identification and Characterization of Histone Modification Gene Families and Their Expression Patterns During Pod and Seed Development in Peanut
Source: Int J Mol Sci. 2025 Mar 13;26(6):2591. doi: 10.3390/ijms26062591 (PMC11942463; doi:10.3390/ijms26062591)

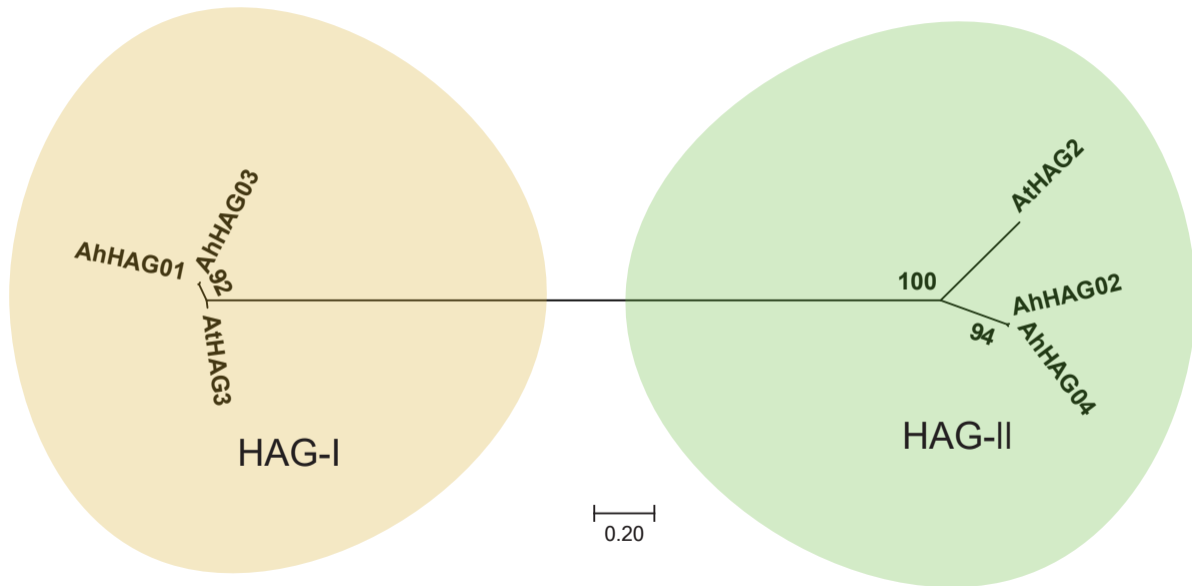

Supplement: Supplementary file 1 [file ijms-26-02591-s001.zip › Figure.S10.At-Ah-HAG-1031.pdf]

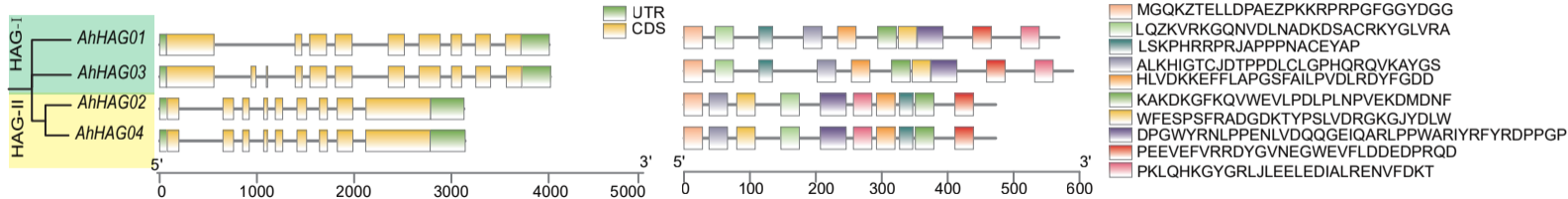

Supplement: Supplementary file 1 [file ijms-26-02591-s001.zip › Figure.S11 Ah-HAG-CDS-MOTIF-1030.pdf]

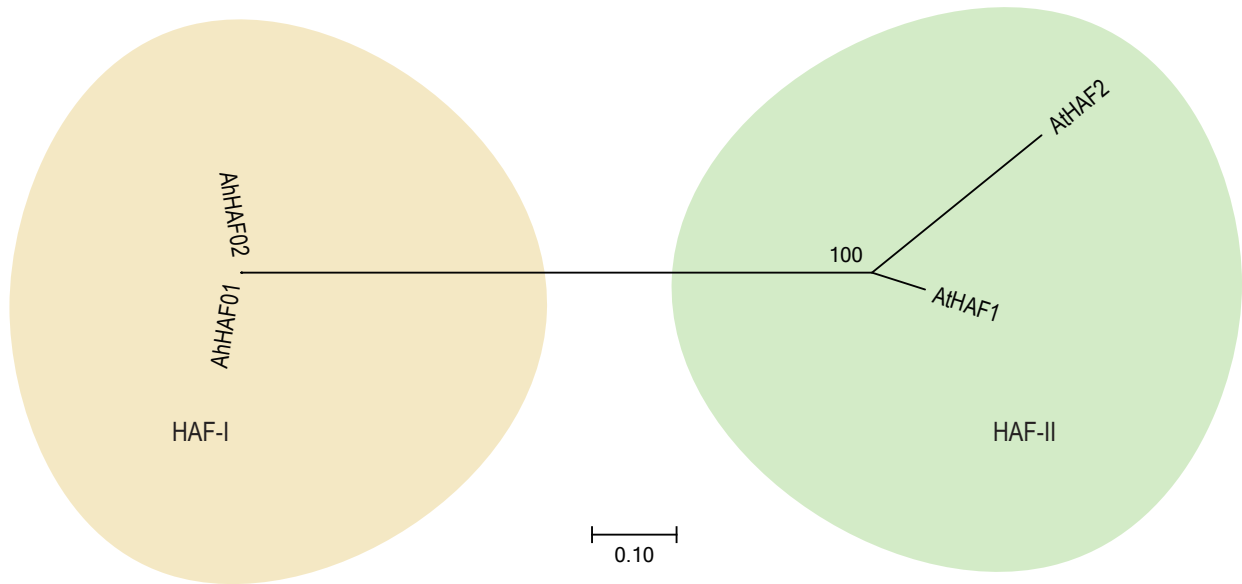

Supplement: Supplementary file 1 [file ijms-26-02591-s001.zip › Figure.S12. At-Ah-HAF-1031.pdf]

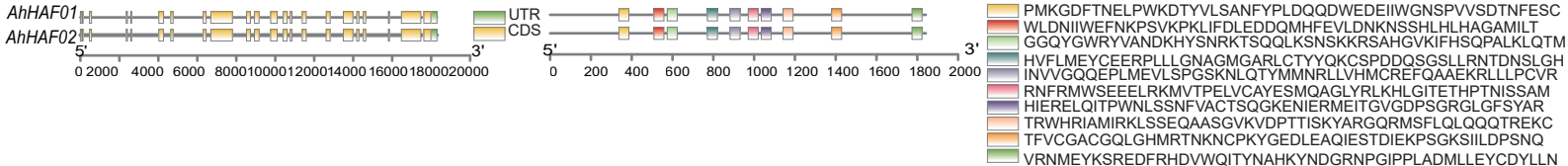

Supplement: Supplementary file 1 [file ijms-26-02591-s001.zip › Figure.S13 Ah-HAF-CDS-MOTIF-1030.pdf]

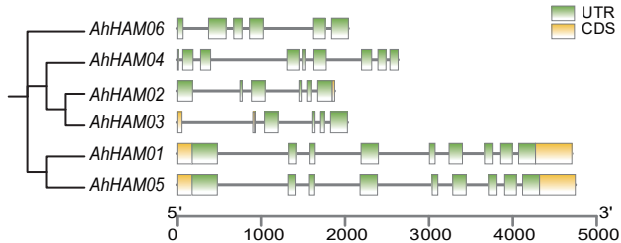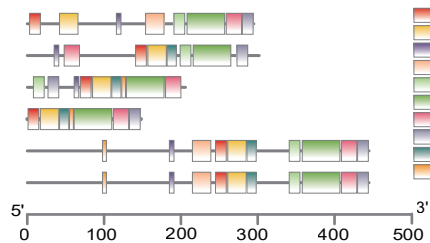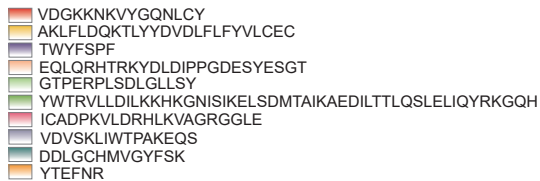

Supplement: Supplementary file 1 [file ijms-26-02591-s001.zip › Figure.S14 Ah-HAM-CDS-MOTIF-1030.pdf]

HDA-II

HDA-III

HDA-I

0.5

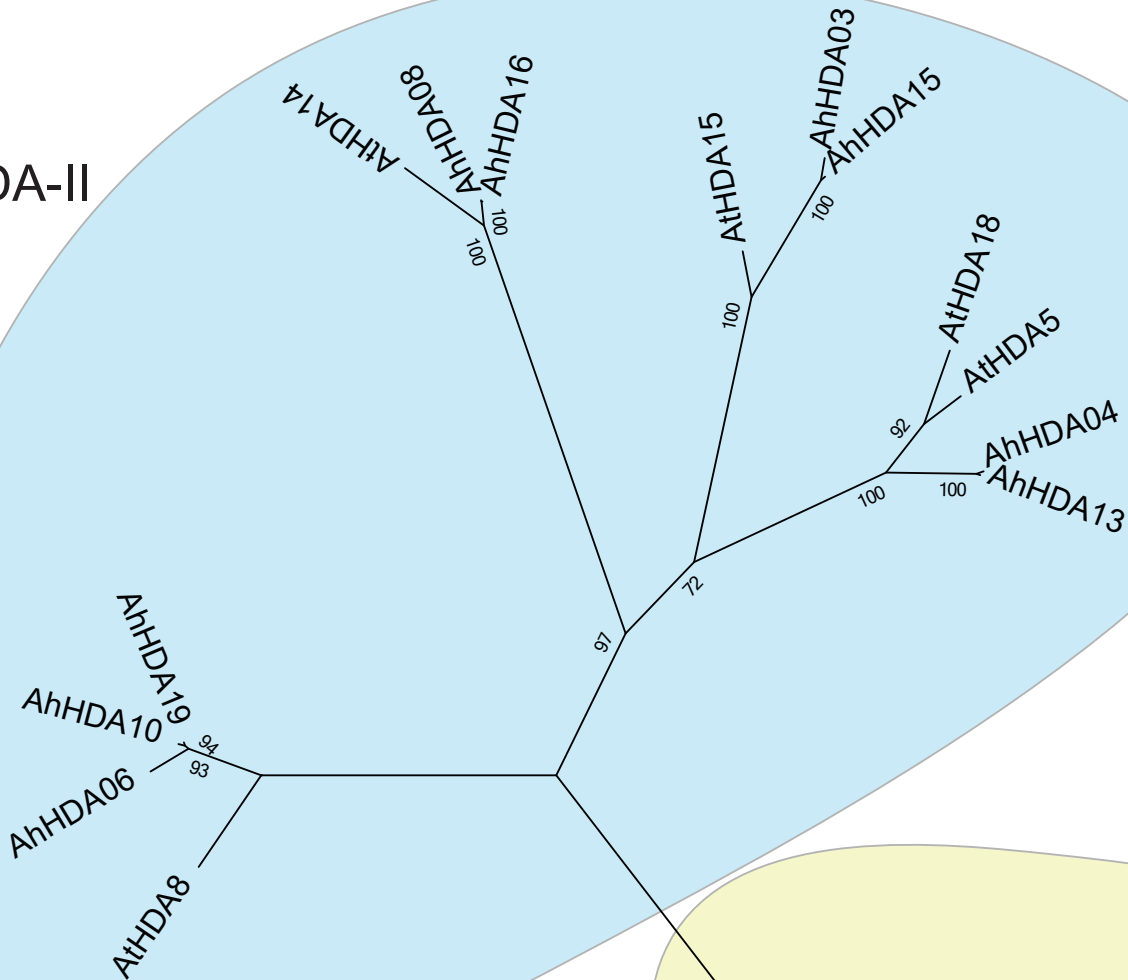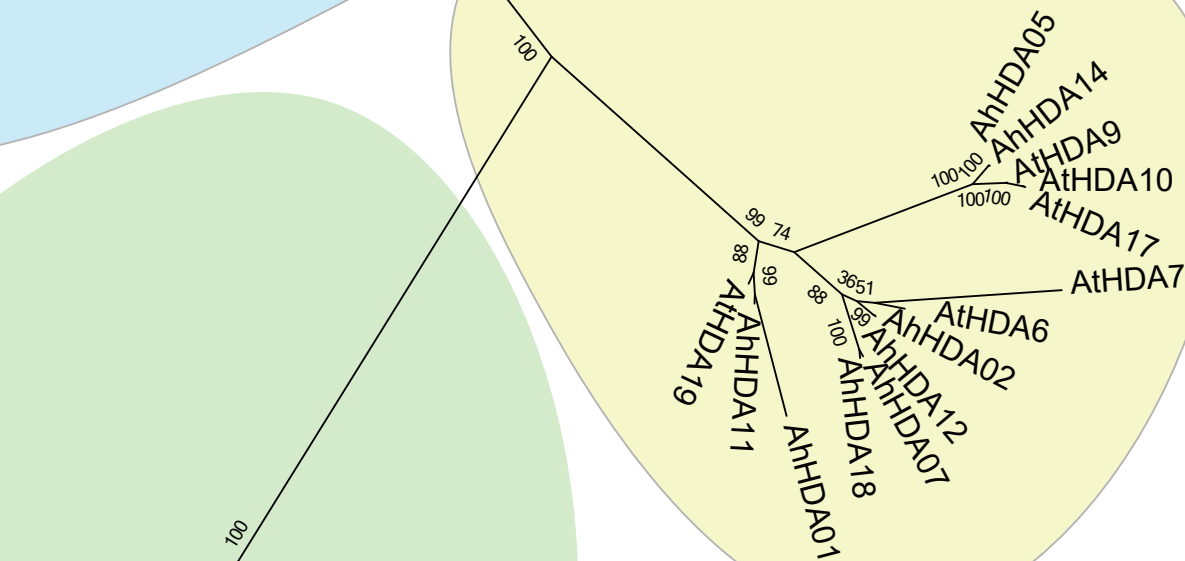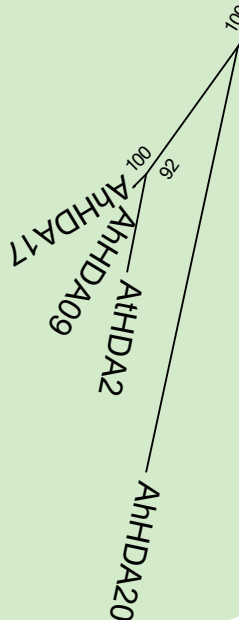

Supplement: Supplementary file 1 [file ijms-26-02591-s001.zip › Figure.S15. At-Ah-HDA-1031.pdf]

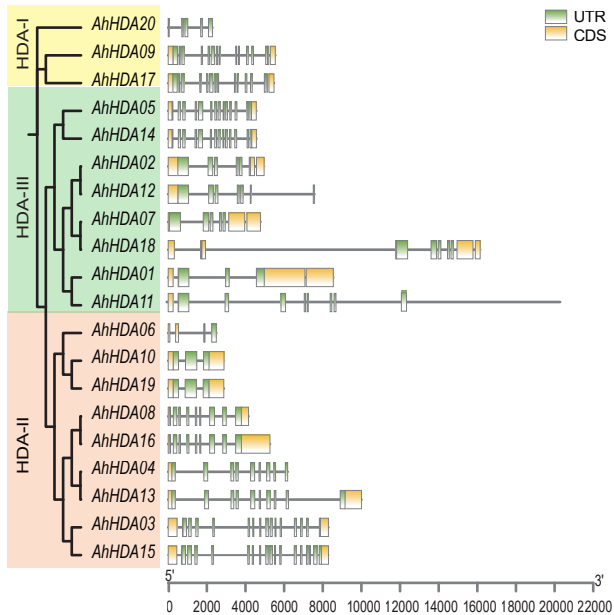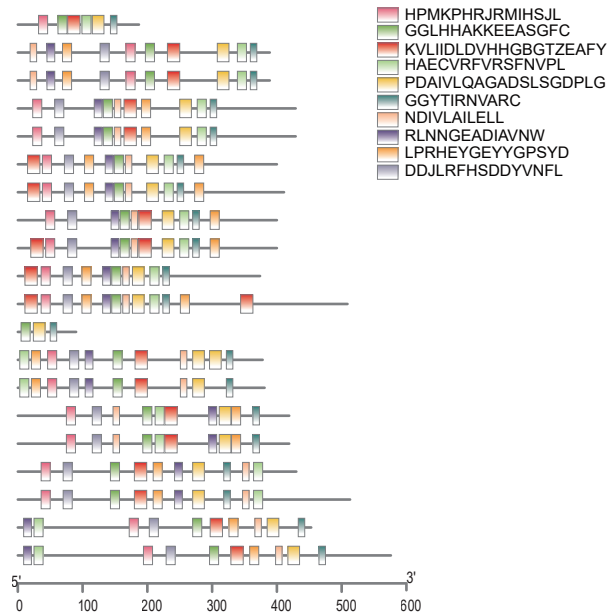

Supplement: Supplementary file 1 [file ijms-26-02591-s001.zip › Figure.S16 Ah-HDA-CDS-MOTIF-.pdf]

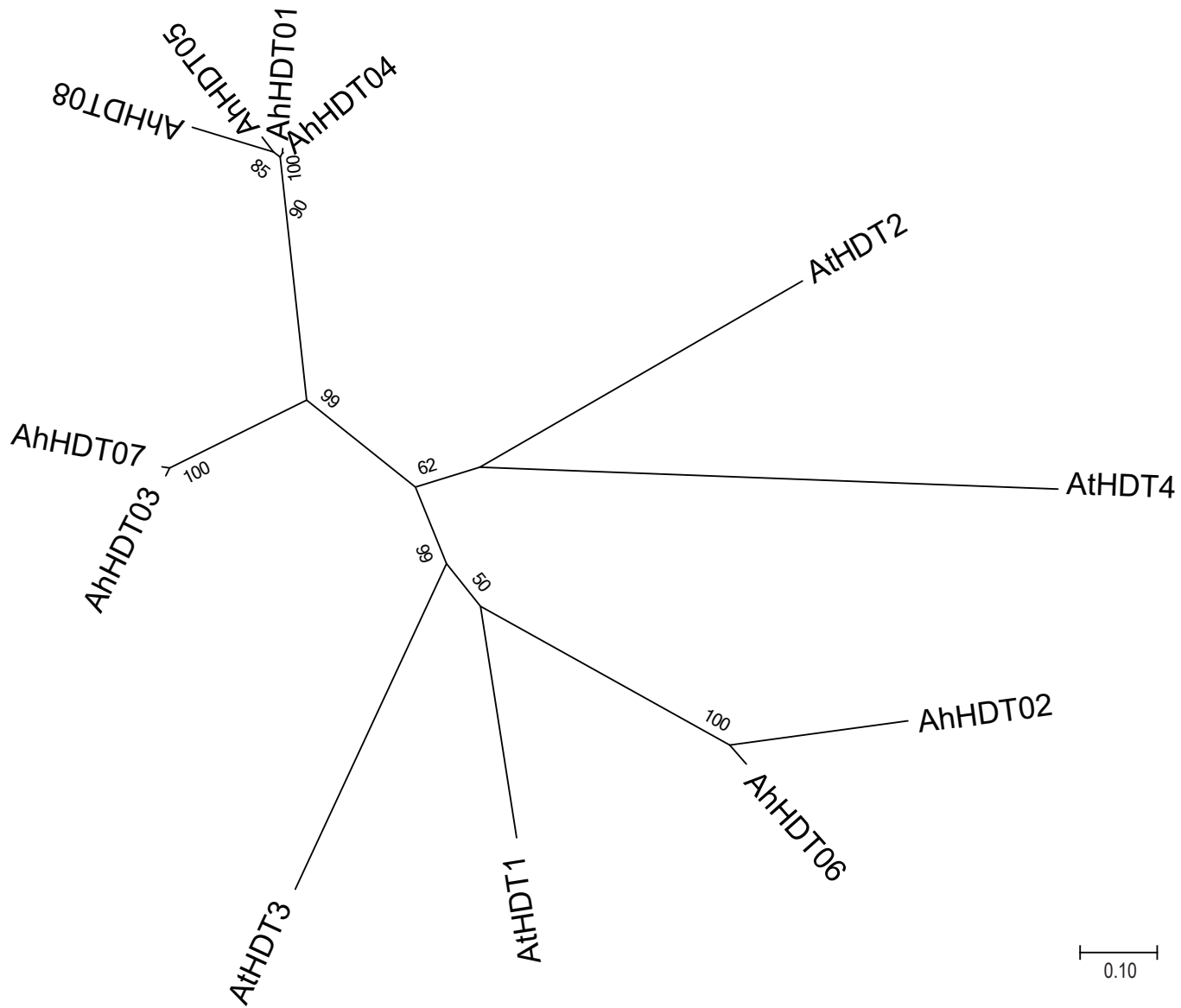

0.10

Supplement: Supplementary file 1 [file ijms-26-02591-s001.zip › Figure.S17. At-Ah-HDT-1031.pdf]

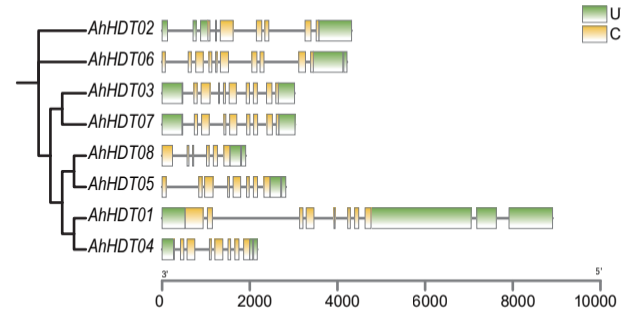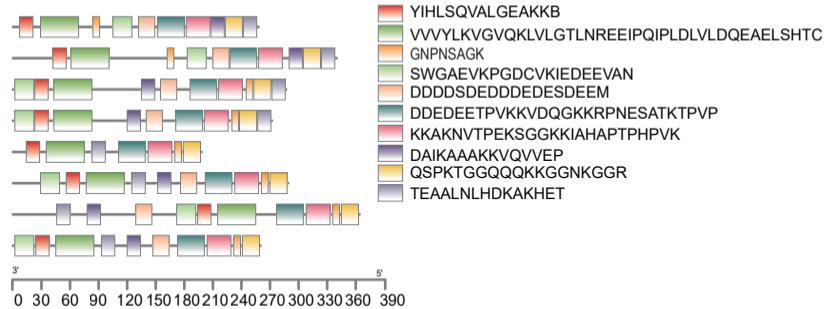

Supplement: Supplementary file 1 [file ijms-26-02591-s001.zip › Figure.S18. Ah-HDT-CDS-MOTIF-.pdf]

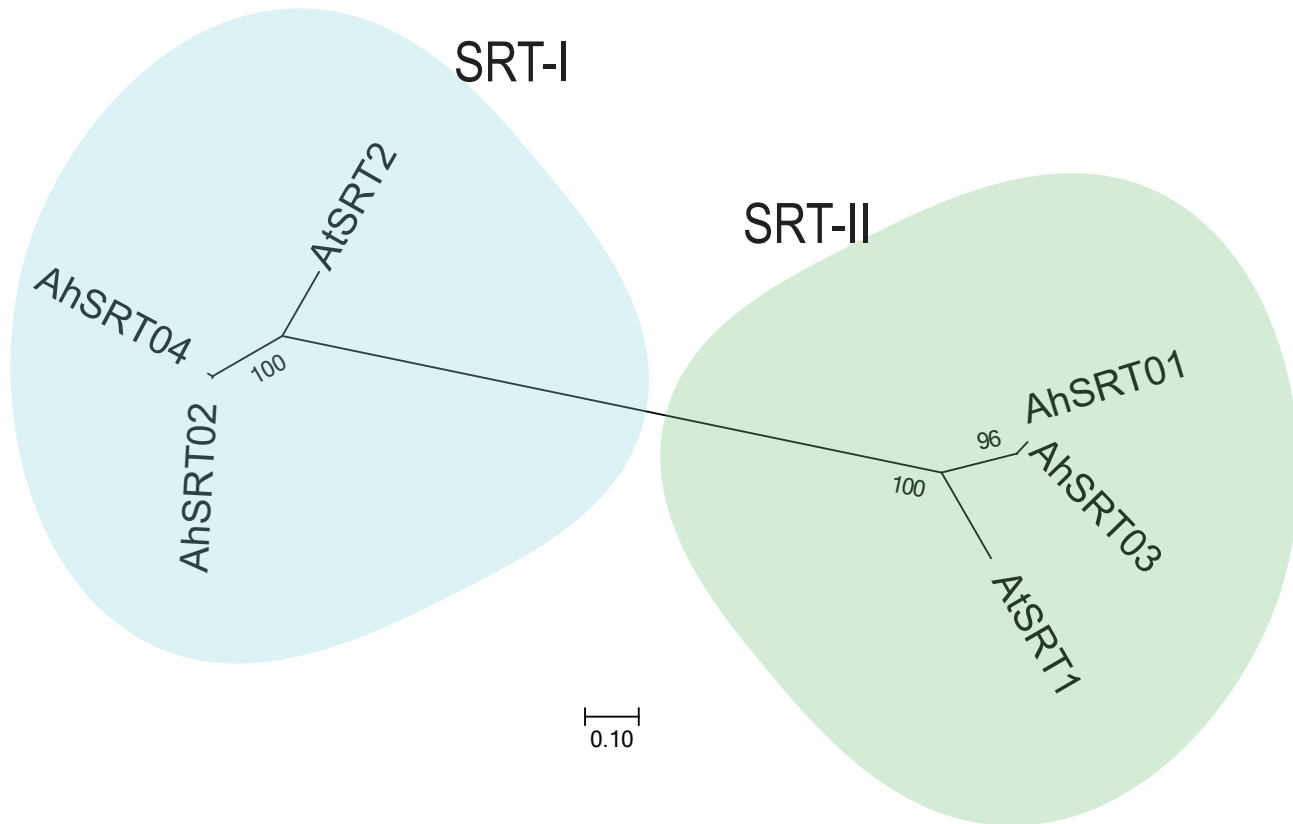

Supplement: Supplementary file 1 [file ijms-26-02591-s001.zip › Figure.S19. At-Ah-SRT-1031.pdf]

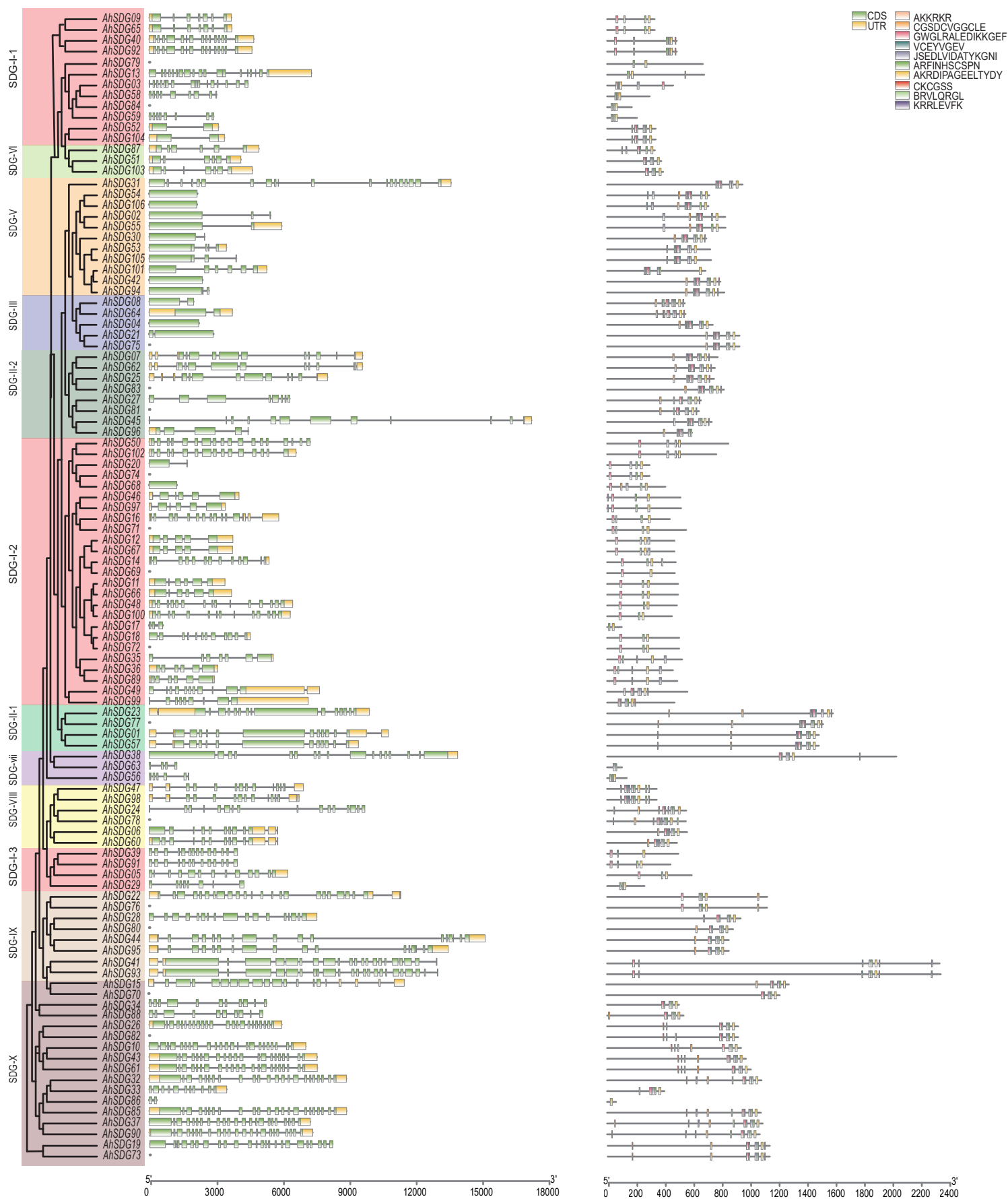

Supplement: Supplementary file 1 [file ijms-26-02591-s001.zip › Figure.S2.AhSDG-CDS-motif-1114-.pdf]

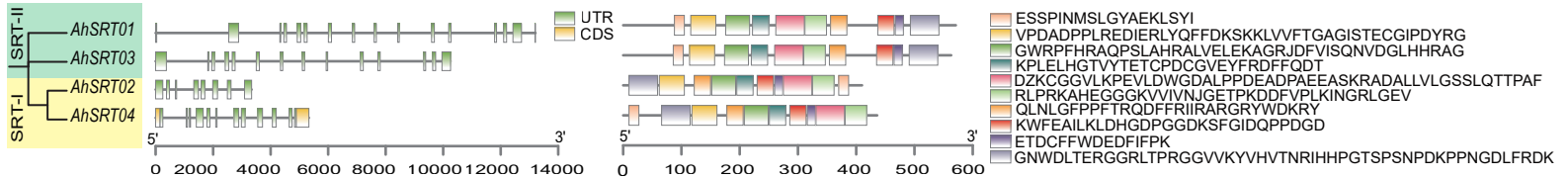

Supplement: Supplementary file 1 [file ijms-26-02591-s001.zip › Figure.S20. Ah-SRT-CDS-MOTIF-.pdf]

## HDMA-I

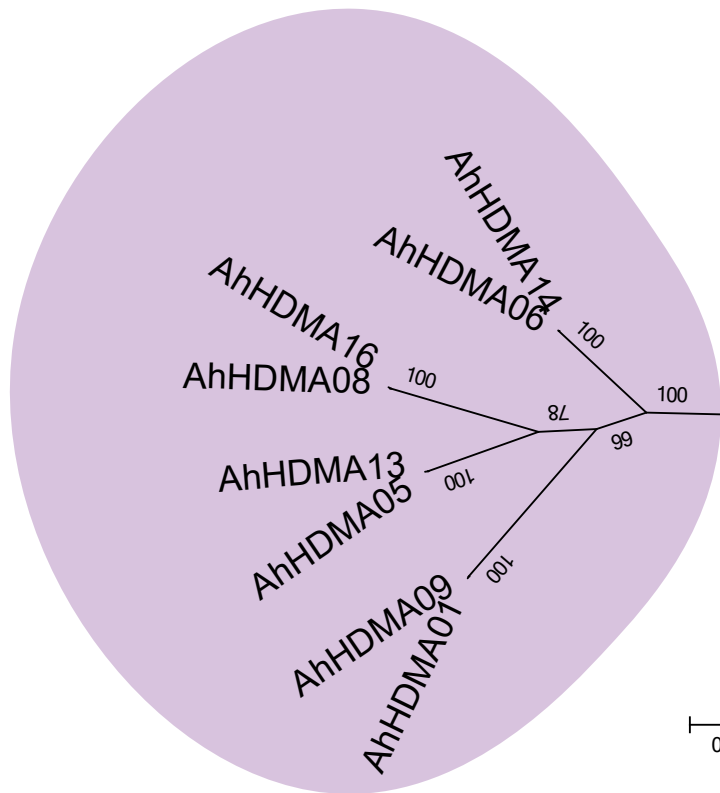

## HDMA-II

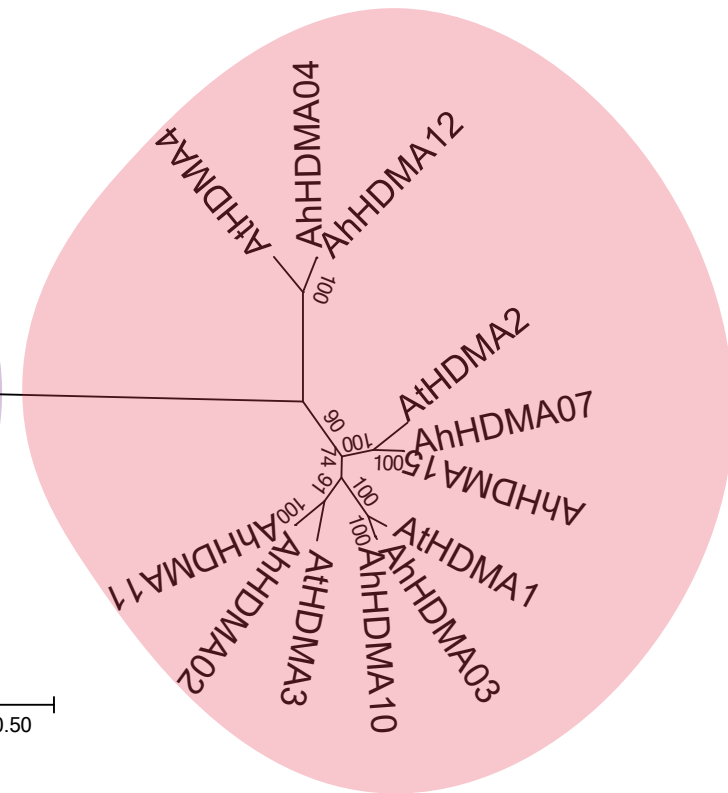

0.50

Supplement: Supplementary file 1 [file ijms-26-02591-s001.zip › Figure.S3 At-Ah-HDMA-11.1.pdf]

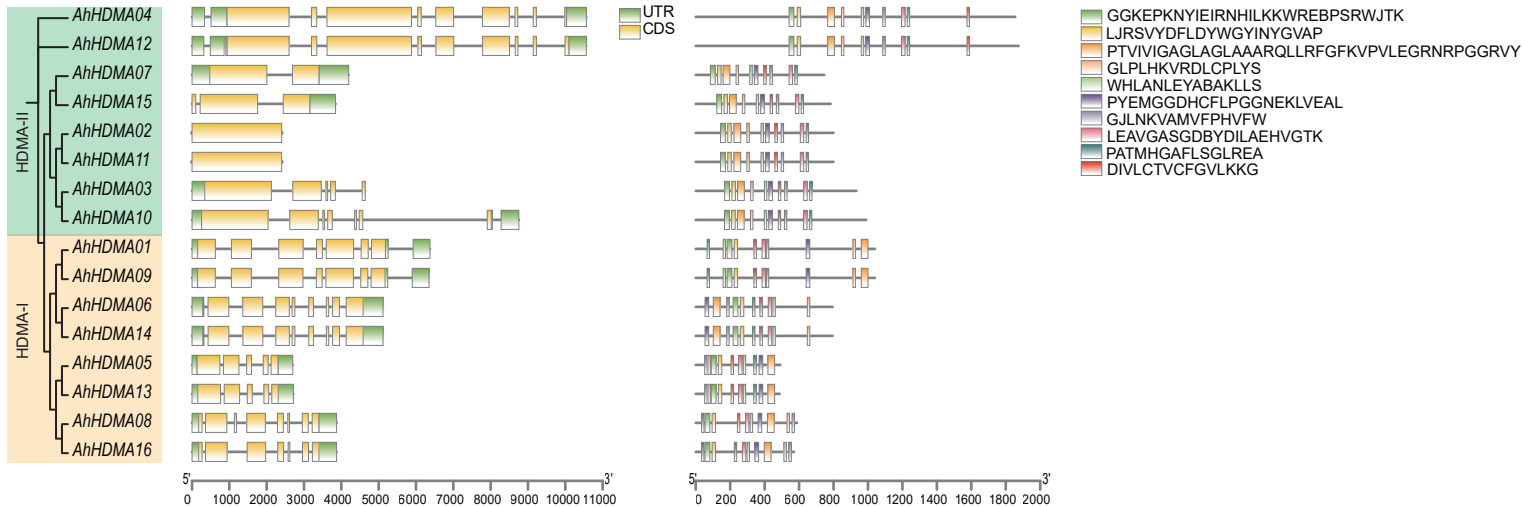

Supplement: Supplementary file 1 [file ijms-26-02591-s001.zip › Figure.S4. Ah-HDMA-CDS-MOTIF-.pdf]

JMJ-I

JMJ-III

JMJ-II

JMJ-IV

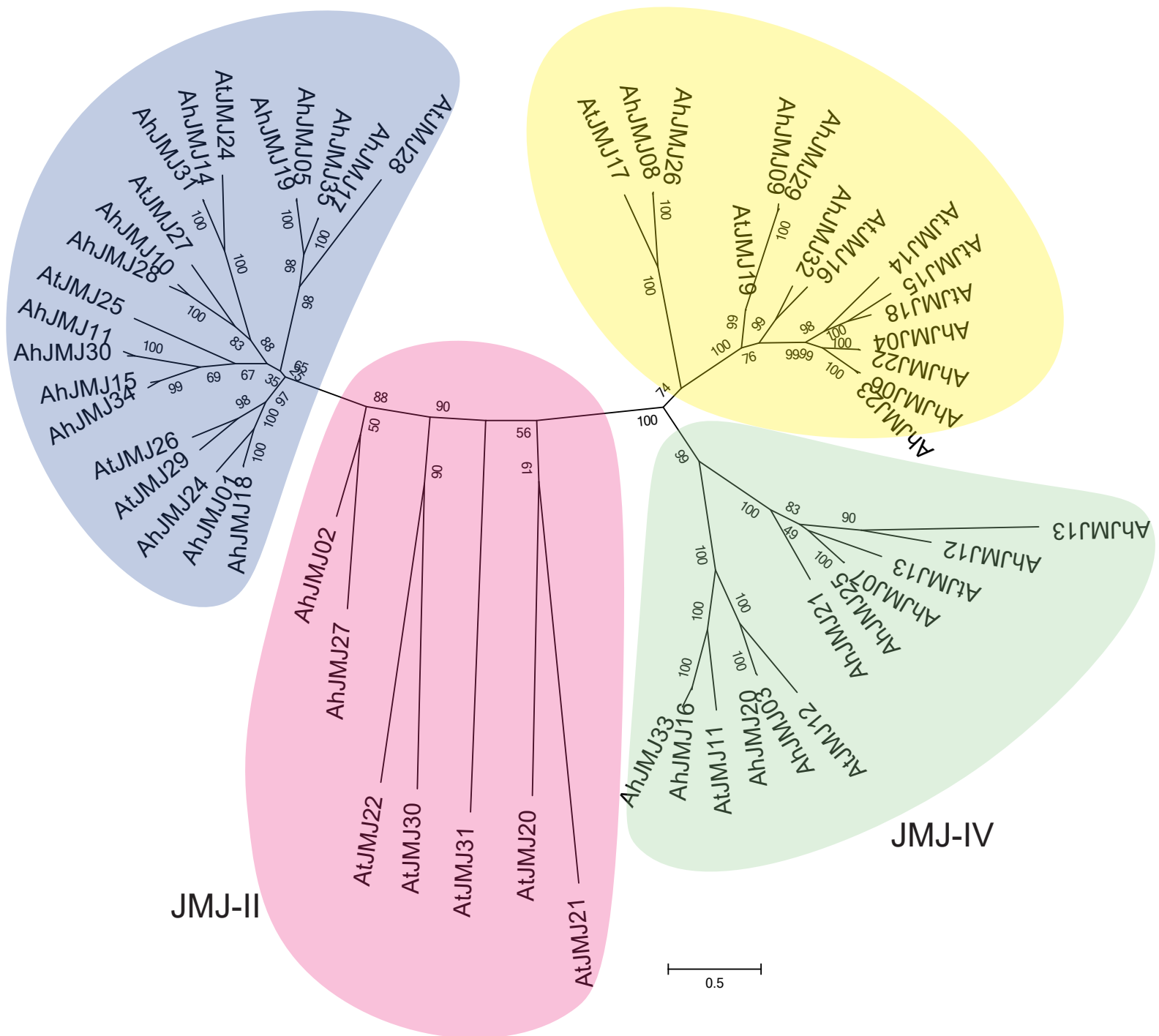

Supplement: Supplementary file 1 [file ijms-26-02591-s001.zip › Figure.S5. At-Ah-JMJ-RA11.1.pdf]

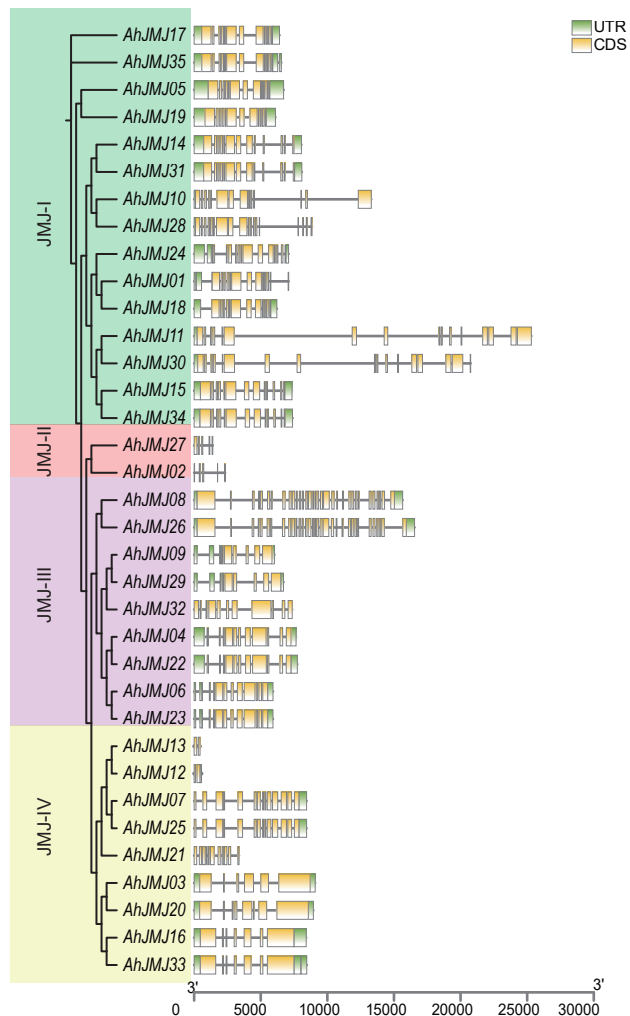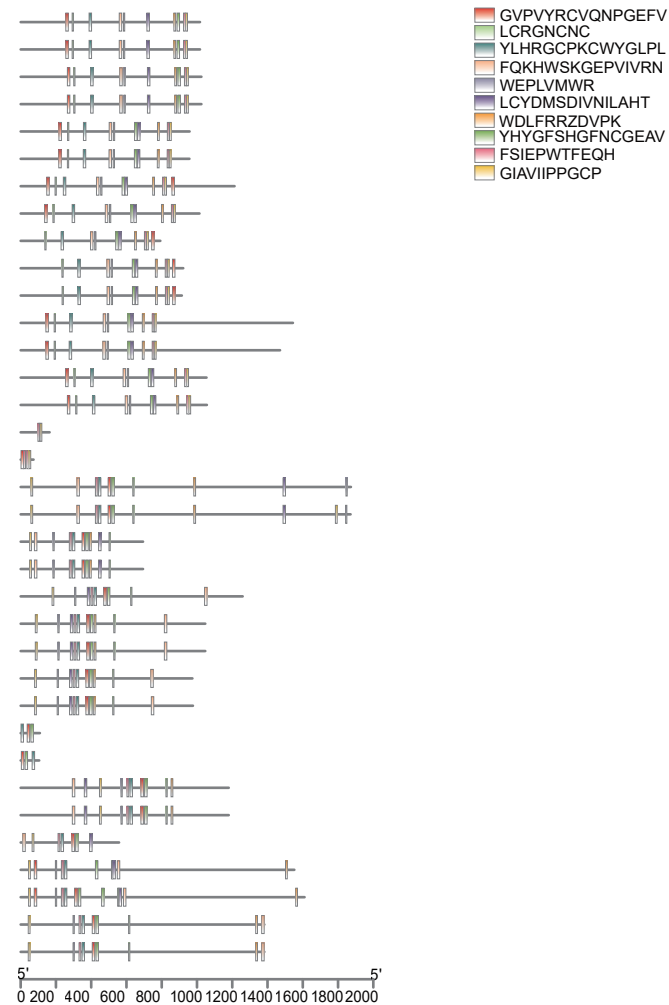

Supplement: Supplementary file 1 [file ijms-26-02591-s001.zip › Figure.S6. Ah-JMJ-CDS-MOTIF-.pdf]

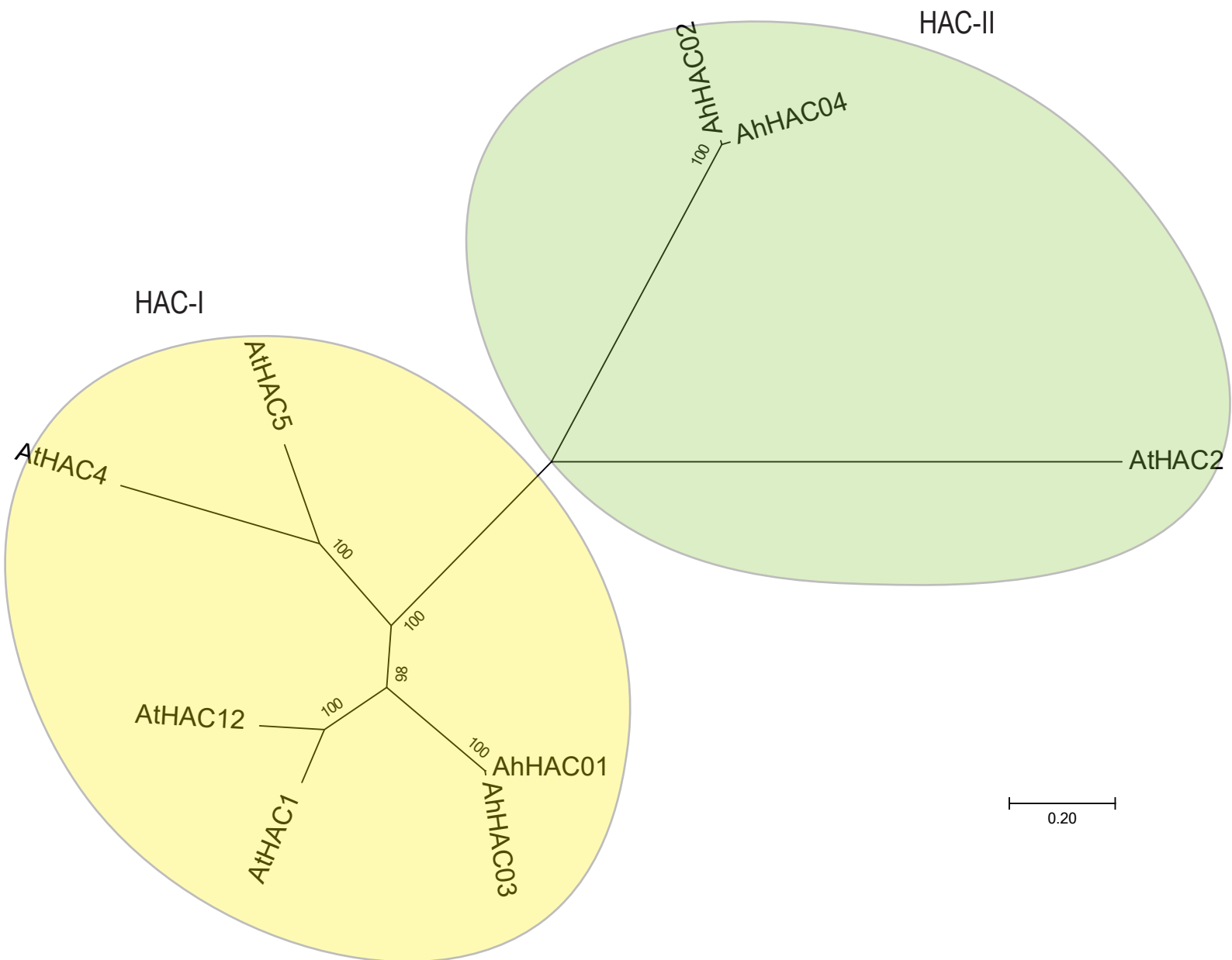

Supplement: Supplementary file 1 [file ijms-26-02591-s001.zip › Figure.S7. At-Ah-HAC-1031.pdf]

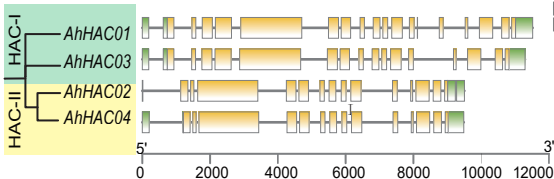

UTR

CDS

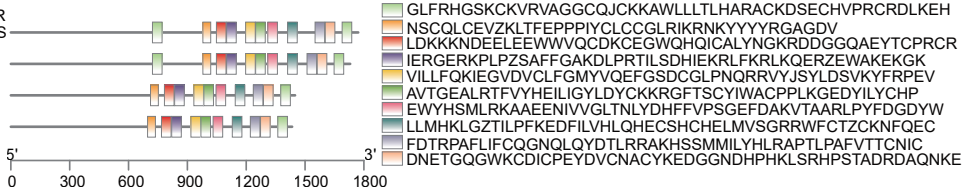

Supplement: Supplementary file 1 [file ijms-26-02591-s001.zip › Figure.S8.Ah-HAC-CDS-MOTIF-1030.pdf]

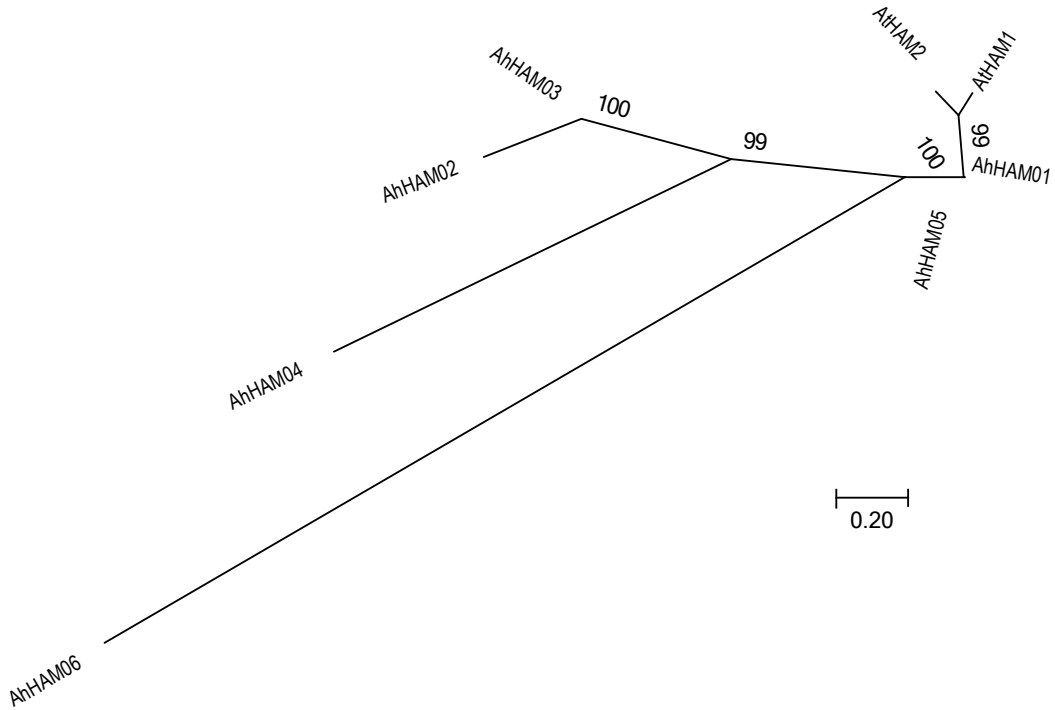

Supplement: Supplementary file 1 [file ijms-26-02591-s001.zip › Figure.S9. At-Ah-HAM-1031.pdf]
